# Bronchoalveolar lavage fluid cytokine profiles in neuroendocrine cell hyperplasia of infancy and follicular bronchiolitis

**DOI:** 10.1186/1750-1172-8-175

**Published:** 2013-11-11

**Authors:** Jonathan Popler, Brandie D Wagner, Heidi Luckey Tarro, Frank J Accurso, Robin R Deterding

**Affiliations:** 1 Georgia Pediatric Pulmonology Associates, P.C., 1100 Lake Hearn Drive, Suite 450, Atlanta, GA 30342, USA; 2 Department of Biostatistics and Informatics, Colorado School of Public Health, University of Colorado Denver, 13123 E. 16th Avenue Aurora, Denver, Colorado 80045, USA; 3 Department of Pediatrics, The Children’s Hospital Colorado, University of Colorado Denver, 13123 E. 16th Avenue, Aurora, Denver, Colorado 80045, USA

**Keywords:** Pediatric interstitial lung disease, Pediatric diffuse lung disease, Children’s interstitial lung disease (chILD), Bronchoalveolar lavage cytokine profiles

## Abstract

**Background:**

Neuroendocrine Cell Hyperplasia of Infancy (NEHI) and Follicular Bronchiolitis (FB) are rare pediatric diffuse lung diseases with poorly understood pathogenesis and similar clinical presentations. We sought to determine if cellular and cytokine profiles in bronchoalveolar lavage fluid (BALF) from subjects with NEHI and FB would differ from pediatric disease controls.

**Methods:**

BALF was obtained from forty-one subjects classified into four disease groups: NEHI, Cystic Fibrosis (CF), other airway disease controls (DC), and FB during clinically indicated procedures. BALF cellular profiles and ten cytokines were measured and values compared across groups using descriptive and nonparametric statistics.

**Results:**

Significant BALF cellular and cytokine differences were seen across all groups. NEHI subjects exhibited the lowest total absolute white blood cell (WBC) levels with a higher percentage of BALF alveolar macrophages compared to controls. NEHI also had lower levels of IL-1β, MIP-1β and IL-8 and FB had higher levels of IL-1ra, G-CSF and VEGF compared to all groups. IL-6 was elevated in CF and FB.

**Conclusions:**

BALF cytokine and cellular profiles differed between NEHI, FB, CF and DC subjects. This pilot data suggests different and distinguishing inflammatory responses in the airway, with the least inflammatory being NEHI. These data could have diagnostic implications.

## Background

Children’s interstitial and diffuse lung diseases (chILD) are a heterogeneous group of rare diseases, characterized by hypoxia, tachypnea, and crackles, in the absence of other diagnoses. Other findings include diffuse infiltrates found on radiographic imaging of the chest and disordered gas exchange [1]. After known causes of lung disease have been ruled out, this constellation of signs and symptoms has been labeled children’s interstitial lung disease (chILD) syndrome [2]. ChILD syndrome includes pulmonary interstitial glycogenosis (PIG), genetic abnormalities of surfactant function, specifically mutations in the surfactant protein B (SP-B), surfactant protein C (SP-C), NKX2.1, and ATP-binding cassette transporter (ABCA3) genes, which predominately involve the interstitium, and neuroendocrine cell hyperplasia (NEHI) and follicular bronchiolitis (FB) which predominately involve the airways [2,3]. These diseases are rare, present similarly, and can often masquerade as other entities, leading to difficult diagnostic dilemmas in the pediatric population. The underlying mechanisms of these disorders are incompletely understood.

Neuroendocrine cell hyperplasia of infancy (NEHI) is a recently described respiratory disorder of unknown etiology characterized clinically by tachypnea, retractions, crackles and hypoxemia [1]. Recognition of NEHI has increased throughout the medical community [1,4]. No mortality has been reported but most children require several years of oxygen therapy and nutritional supplementation [5]. Treatment with glucocorticoids has not been effective [4]. NEHI often presents with a characteristic appearance on high-resolution computed tomography (HRCT). The pattern consists of ground glass opacities (GGOs) most commonly occurring in the right middle lobe and lingula; additionally, mosaic air trapping is also seen [6,7]. Infant pulmonary function testing in NEHI has shown evidence of small airway obstruction and air trapping [4,8,9]. The term “NEHI syndrome” is used to indicate a diagnosis of NEHI without lung biopsy, based on a consistent clinical presentation and HRCT findings [10,11]. The diagnostic gold standard is lung biopsy demonstrating increased numbers of bombesin–immunopositive pulmonary neuroendocrine cells (PNECs) within bronchioles and alveolar ducts without evidence of other abnormalities, and limited or absent inflammation [2].

Despite improved clinical characterization, the underlying etiology for NEHI is unknown and a genetic defect has not been reported. However, multiple cases of NEHI have been found within several families, suggesting the mechanism of disease may be related to a genetic etiology [12].

Follicular bronchiolitis (FB) is a rare chILD disorder with a paucity of pediatric literature. In a series of five FB patients, patients presented with cough, crackles and respiratory distress features that overlap with NEHI. There was little glucocorticoid responsiveness (13). Histologic findings were characterized by follicular lymphoid hyperplasia around the bronchus and between the bronchiole and pulmonary artery in all patients. These follicles have germinal centers and frequently compress the bronchial lumen. A concentric ring of lymphocytes surrounded the bronchioles [13].

In patients with chILD syndrome, histopathologic data suggest that inflammation may be important to the underlying pathogenesis of many these disorders, except in NEHI where few signs of inflammation are seen histologically. As BALF is less invasive than lung biopsy, we elected to study cellular and cytokine profiles. Although BALF cytokine profiles have been investigated in adult interstitial lung disease (ILD), to our knowledge no similar investigation has been done within the chILD population [14].

We hypothesized that BALF cellular and cytokine profiles in NEHI and FB would differ from profiles in pediatric airway disease control subjects. Furthermore we believed that that NEHI would show limited pro-inflammatory cytokine elevation in contrast to FB and airway disease controls based upon lung pathology that has shown limited inflammation and clinical reports of poor response to glucocorticoids.

## Methods

### Subjects

The study was designed as a single center descriptive study to measure cytokine levels in the BALF of children with NEHI and FB compared to disease controls. These were clinically indicated samples collected from 1998 to 2009. As NEHI and FB are felt to be predominately airway related diseases, we chose airway disease control subjects with cystic fibrosis (CF), bronchiectasis (non-cystic fibrosis), bronchiolitis obliterans, or suspected aspiration syndromes as the comparison group.

Within the NEHI group, both patients with biopsy-proven NEHI (n= 11), as well as patients with NEHI syndrome (n=11) were combined. NEHI syndrome subjects were those who had not undergone lung biopsy, but had symptoms consistent with NEHI, including tachypnea, crackles, and desaturation. All NEHI syndrome patients underwent HRCT with images reviewed by a radiologist experienced in recognizing NEHI and all had findings consistent with NEHI including ground glass opacities (GGO) and air trapping [10-12].

Subjects with FB were diagnosed by lung tissue review by a pediatric pathologist experienced in identifying the typical appearance of FB. Disease controls were confirmed by appropriate clinical presentation and confirmatory testing. CF was diagnosed by sweat test and genotyping. The diagnosis of non-cystic fibrosis bronchiectasis and bronchiolitis obliterans were confirmed by chest CT. Subjects with suspected aspiration syndromes had confirmatory testing with swallowing evaluations with oropharyngeal motility studies.

Disease control subjects also underwent clinically-indicated bronchoscopy and lavage. If multiple longitudinal dates for BALF sample collection were recorded from a subject, the first BALF sample with an acceptable volume percentage was used. Published values for cell counts and cytology differentials in the BALF of healthy, non-wheezing children were also used as normal control reference ranges [15,16].

The study was approved by the University of Colorado Denver Institutional Review Board. Informed consent was obtained from all subjects. If the patient was a minor, informed consent was obtained from the subject’s legal guardian. In subjects aged 12 to 17 years, informed assent was obtained.

### Technique for obtaining BALF

BALF was obtained in the following manner. After induction of general anesthesia, a flexible bronchoscope was passed into the airway via an endotracheal tube or laryngeal mask airway. Lidocaine was placed at both the vocal cords and carina. The area of lavage varied, and was selected by the pulmonologist performing the procedure. In general, lavage was performed in areas where mucus had collected, in areas where infiltrate was seen on chest x-ray or chest computed tomography scan (CT scan) or from the right middle lobe. Typically three lavages were performed using sterile nonbacteriostatic saline at room temperature and pooled. Each lavage aliquot consisted of 1 milliliter per kilogram of body weight, with a maximum of 30 milliliters of normal saline per lavage. After BALF collection, any excess fluid not required for clinical laboratory testing was processed for research.

Collected BALF was processed in a standard manner. It was initially centrifuged for 10 minutes at 1200 revolutions per minute (rpms) and 4 degrees Celsius. The pellet was saved, and the supernatant was spun again for 20 minutes at 4700 rpms and 4 degrees Celsius. The second pellet was saved. This supernatant was split in half, with half stored 0.5 mL aliquots at −70 degrees Celsius. The other half of the supernatant was treated with the protease inhibitors Phenylmethylsulfonyl Fluoride (PMSF), and Ethylenediaminetetra acetic acid (EDTA) to inhibit protease activity. The samples are frozen at −70°C for further study.

### Bronchoalveolar lavage cell counts and differentials

Collected BALF was centrifuged to separate fluid from cells. The cell pellet was resuspended and cell counts and differentials were determined. Results were expressed as viable cells/mL of lavage fluid.

### Microbiology

Bacterial culture and viral culture, Direct Fluorescent-Antibody (DFA; until 2007) and polymerase chain reaction (PCR after 2007) were performed on the collected BALF. Common organisms were screened for, including *Staphylococcus* and *Streptococcal* species, *Haemophilus influenzae*, *Moraxella catarrhalis*, respiratory syncytial virus, influenza A and B, parainfluenza 1, 2, and 3, and rhinoviruses. Human metapneumovirus has been screened for since 2007.

### Normalization factors

Normalization of BALF was done as recommended by consensus guidelines to report the percentage of volume recovered compared to volume instilled. The BALF recovered was required to be at least 30% of the volume instilled [15-17].

### Cytokine analysis

Cytokine profiles were analyzed using the Fluorokine® MultiAnalyte Profiling (MAP) Cytokine Assay (R&D systems) and the Luminex® 200™ analyzer, which measures multiple analytes in a 50 microliter aliquot of processed BALF. This system is a dual laser, flow-based sorting and detection platform, which uses analyte-specific antibodies pre-coated onto color-coded microparticles to measure 1–18 possible cytokines. Microparticles are read using the Luminex 200 analyzer. One laser is microparticle-specific and determines which cytokine is being detected. The other laser determines the magnitude of the phycoerythrin-derived signal, which is in direct proportion to the amount of analyte bound. The individual cytokines are quantified against an 8-point calibration curve run with each plate.

### Statistical analysis

Descriptive statistics were calculated using medians, ranges, and interquartile ranges (IQR) where specified, for continuous variables and percentages for categorical variables. Kruskal – Wallis nonparametric tests were used to compare cytokine values across the four disease groups. Pairwise comparisons from nonparametric tests were completed if the overall p-value comparing all 4 groups was < 0.05. Nonparametric canonical discriminant analysis (CDA) was performed to study the differences between groups with respect to all BALF variables simultaneously. CDA is a multivariate method similar to Principal Component Analysis (PCA) yet differs in that we have prior knowledge of the groups. A normal kernel density with a radius of 0.4 and the individual within-group covariance matrices were specified for the CDA. Crossvalidation was used to estimate classification error rates. All analyses were performed using SAS version 9.3 software (SAS Institute Inc.: Cary, NC, 2011).

## Results

### Subject population

BALF samples from 41 patients with 8 different clinical diagnoses were included in this investigation. The disease groups consisted of 22 NEHI subjects, 6 FB subjects and disease controls including 7 CF disease controls, and 6 non-CF disease controls (2 Bronchiolitis Obliterans, 1 Primary Ciliary Dyskinesia, 1 recurrent aspiration, 1 non-cystic fibrosis bronchiectasis and 1 immunodeficiency). The median age for the subjects at the time of BALF collection was 1.5 years ranging between 1 month and 11 years old; the CF group had slightly older subjects. The CF and non-CF disease controls tended to be mostly males, while the NEHI and FB groups consisted of a more even gender mix. BALF volume percent returned was similar across groups. Forty-two percent of BALF samples had positive cultures. As expected the CF group had the highest percentage of positive cultures (86%) followed by the FB (50%) and NEHI (32%) (Table 1).

**Table 1:** Subject demographics

	**NEHI (N = 22)**	**FB (N = 6)**	**CF (N = 7)**	**Non-CF DC (N = 6)**
Age at BAL in years, med (min - max)	1 (0.1 - 6)	4 (1–11)	2 (1–6)	6 (0.3 - 9)
Male, N (%)	13 (59%)	3 (50%)	5 (71%)	4 (67%)
Volume percent returned, med (min - max)	57% (32–78)	56% (33–75)	48% (33–53)	47% (33–52)
Culture				
Positive for Potential Bacterial Pathogens	7 (32%)	3 (50%)	6 (86%)	1 (17%)
Types of pathogens	*Staphylococcus aureus* (2)	*Haemophilus influenzae* (2)	*Staphylococcus aureus *(2)	Oxidase negative non-lactose fermenter (1)

	*Haemophilus influenzae* (1)	*Group A β-hemolytic streptococcus* (1)	*Haemophilus influenzae* (2)	
			*Moraxella catarrhalis* (1)	
	*Moraxella catarrhalis* (3)		*Stenotrophomonas maltophilia* (1)	
	*Pseudomonas aeruginosa* (non mucoid) (1)		*Aspergillus Fumigatis* (2)	
			*Scedosporium*	
			*Apiospermum* (1)	
	*Serratia marcescens* (1)			
Virus	CMV (1)	CMV (1)		
		Human Metapneumovirus (1)		

### Cell count and differential

**Figure 1:**
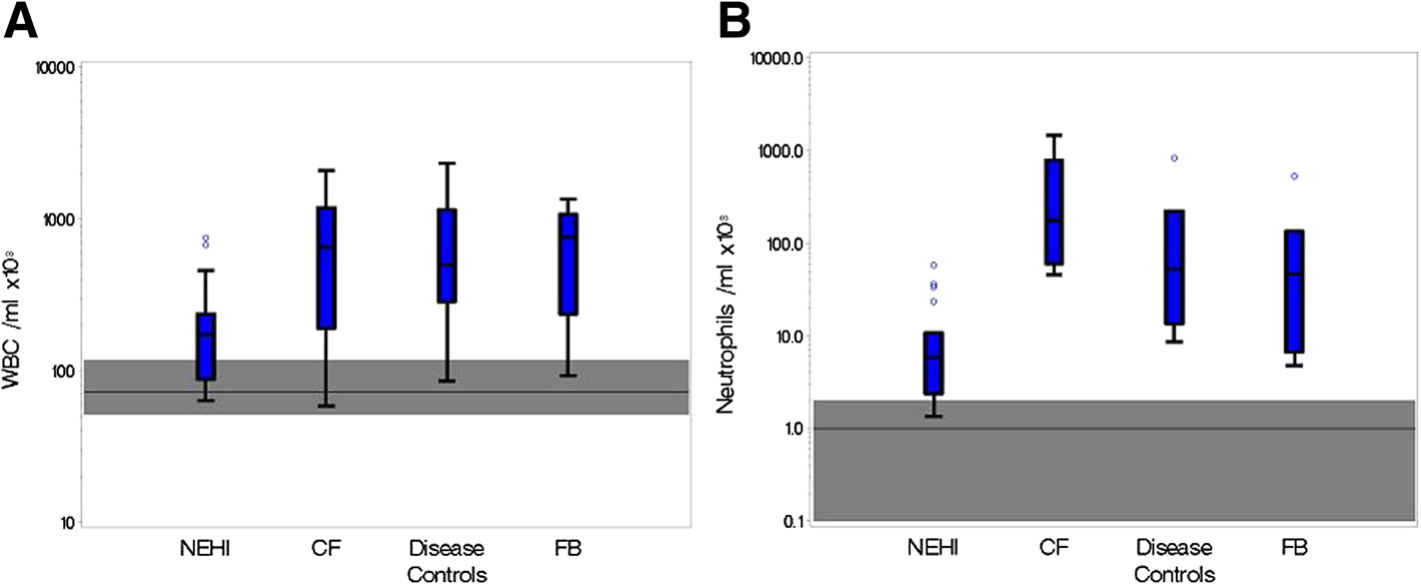
**Comparison of WBC and neutrophil counts across disease groups.** Boxplots displaying the distribution of absolute WBC counts **(A)** and Neutrophil counts **(B)** across the 4 disease groups. The middle line corresponds to the median; the box indicates the 25th and 75th percentiles; the whiskers are drawn from the box to the most extreme point within 1.5 interquartile ranges and any value more extreme than this is marked with a point. The horizontal reference line and shaded area correspond to the median and normal range of values in healthy patients from previously published data.

The absolute number of BALF WBCs in NEHI was significantly lower compared to all other groups but higher than historical normal levels reported in the literature [13] (Figure 1). The percentage of BALF neutrophils in NEHI was also significantly lower compared to all other groups. Conversely, CF had the highest levels of BALF neutrophils across all groups (p-value <0.01). The percentage of BALF alveolar macrophages in NEHI was similar to FB but higher than CF and DC. The percentage of lymphocytes and eosinophils were not dramatically different between groups (Table 2).

**Table 2:** BALF cell count and differential for NEHI, FB, CF and DC

**Med**	**NEHI**	**FB**	**CF**	**Non-CF DC**	**KW**
**(p25, p75)**	**(N = 22)**	**(N = 6)**	**(N = 7)**	**(N = 6)**	**p value**
WBC	173 (88, 236)	761 (238, 1070)	650 (191, 1200)	500 (287, 1150)	0.02
RBC	16 (2, 40)	175 (61, 267)	33 (7, 1100)	150.5 (22, 326)	0.07
Segs %	2.5 (1, 5)	10 (2, 43)	67 (32, 79)	13 (3, 34)	<.01
EOS %	0 (0, 0)	0.5 (0, 1)	0 (0, 0)	0 (0, 1)	0.44
Lymphocytes %	7 (2, 10)	7.5 (2, 15)	1 (0, 3)	9 (5, 11)	0.07
Macrophages & Monos %	84 (76, 90)	77 (43, 88)	30 (14, 61)	63 (55, 86)	<.01
Epithelium %	1.5 (0, 6)	0 (0, 1)	0 (0, 2)	3.5 (0, 9)	0.21

### Cytokine analyses

**Figure 2:**
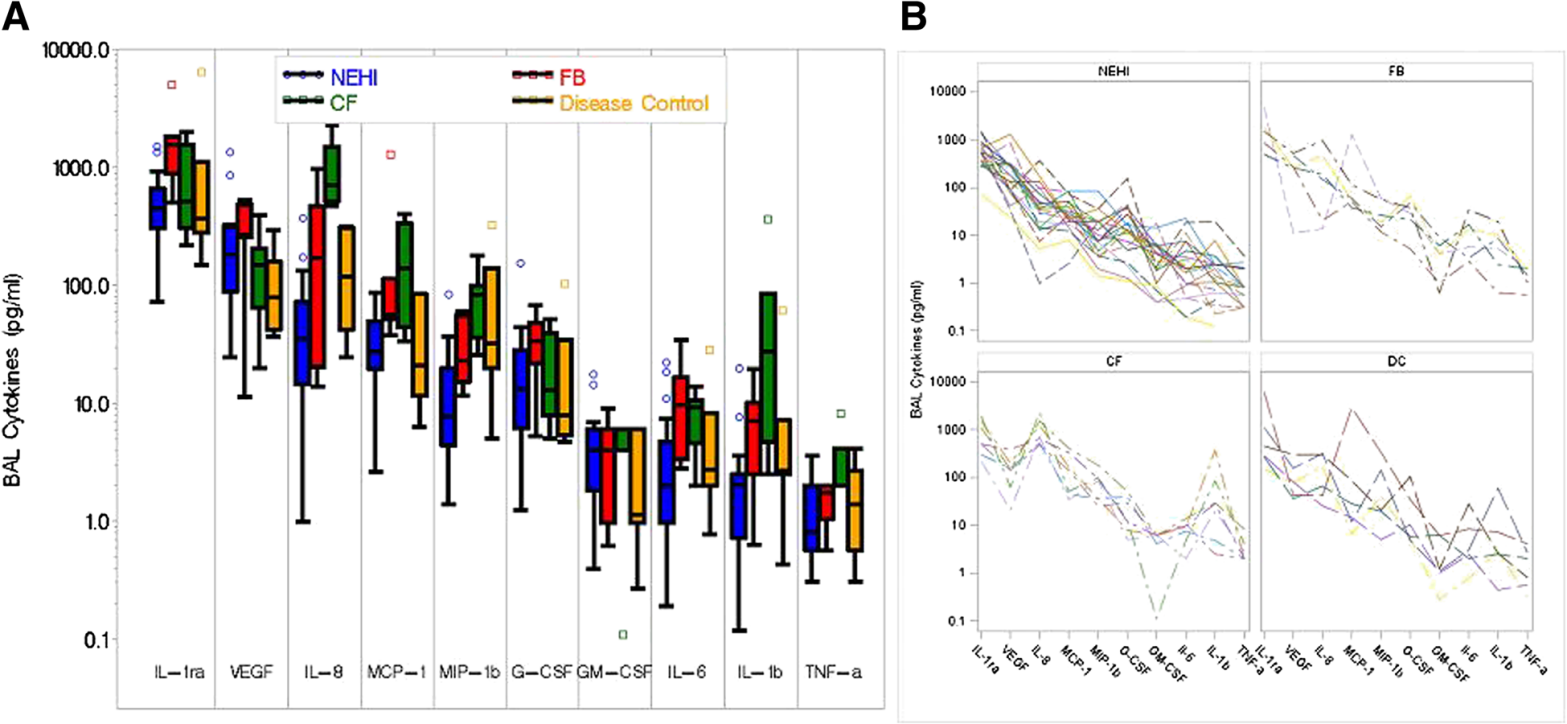
**Comparison of cytokine levels across disease groups.** Cytokine profiles identified in BALF of NEHI, FB, CF and Disease Controls all values expressed as pg/mL. **A)** Shows quantities on the log scale for each cytokine by group with box whiskers. The cyokines are ordered by overall median value. The middle line corresponds to the median; the ends of the box indicate the 25th and 75th percentiles; the whiskers are drawn from the box to the most extreme point within 1.5 interquartile ranges and any value more extreme than this is marked with a point. **B)** Patient specific cytokine profiles separately by group. Cytokine values within a subject are connected by lines.

The following list of cytokines were significantly different between the four disease groups; IL-1β, IL-8, MIP-1β, MCP-1 and IL-6 (Table 3). In NEHI, IL-1β, IL-8 and MIP-1β levels were considerably lower (the 75th percentile was less than the median) than in both disease controls and FB. In FB, IL-1ra, VEGF and G-CSF levels were noticeably higher (the 25th percentile was greater than the median) than in both disease controls and NEHI (Figure 2A, 2B). IL-6 was elevated in both CF and FB. VEGF, G-CSF, and GM-CSF levels between groups did not differ dramatically.

**Table 3:** Cytokines identified in BALF of NEHI, FB, CF and disease controls: all values expressed as pg/mL

**Median**	**NEHI**	**FB**	**CF**	**Non-CF DC**	**KW**
**(IQR)**	**(N = 22)**	**(N = 6)**	**(N = 7)**	**(N = 6)**	**p value**
IL-1β^1^	2.1 (0.7, 2.5)	7.0 (2.5, 10.1)	27.7 (4.7, 85.9)	2.7 (2.5, 7.2)	<0.01
IL-1ra^1^	449.8 (306.6, 663.2)	1558.9 (885.9, 1843.1)	511.8 (308.7, 1567.1)	369.3 (278.6, 1107.4)	0.10
IL-6^1^	2.0 (1.0, 4.7)	9.7 (3.3, 16.5)	9.2 (4.6, 10.6)	2.8 (2.0, 8.4)	0.04
IL-8^1^	35.5 (14.5, 71.7)	171.3 (20.4, 477.7)	705.2 (521.1, 1504.2)	120.0 (42.3, 306.6)	<0.01
G CSF	13.2 (6.1, 27.9)	33.5 (21.8, 48.6)	13.1 (7.9, 39.4)	8.0 (5.4, 34.5)	0.39
GM CSF	4.0 (1.8, 6.0)	4.0 (1.0, 6.0)	6.0 (4.0, 6.0)	1.1 (1.0, 6.0)	0.59
MCP 1^1^	27.6 (19.6, 49.6)	56.1 (51.9, 113.2)	138.2 (43.9, 335.8)	21.1 (11.6, 84.8)	0.01
MIP 1β^1^	7.7 (4.4, 20.0)	22.8 (15.4, 55.3)	83.3 (36.0, 100.2)	31.9 (20.0, 140.3)	<0.01
TNF α	0.8 (0.6, 2.0)	1.7 (1.0, 2.0)	2.0 (2.0, 4.1)	1.4 (0.6, 2.7)	0.06
VEGF	182.1 (89.3, 317.5)	270.5 (254.7, 486.6)	149.2 (64.2, 206.4)	79.6 (42.3, 159.4)	0.30

^1^Significant difference (p < 0.05) observed between NEHI and FB.

Within the NEHI group of patients, cytokine levels were investigated between biopsy proven (n =11) and NEHI syndrome (n =11) patients, culture positive/negative classification, as well as change with age. These preliminary evaluations did not yield striking differences or trends.

**Figure 3:**
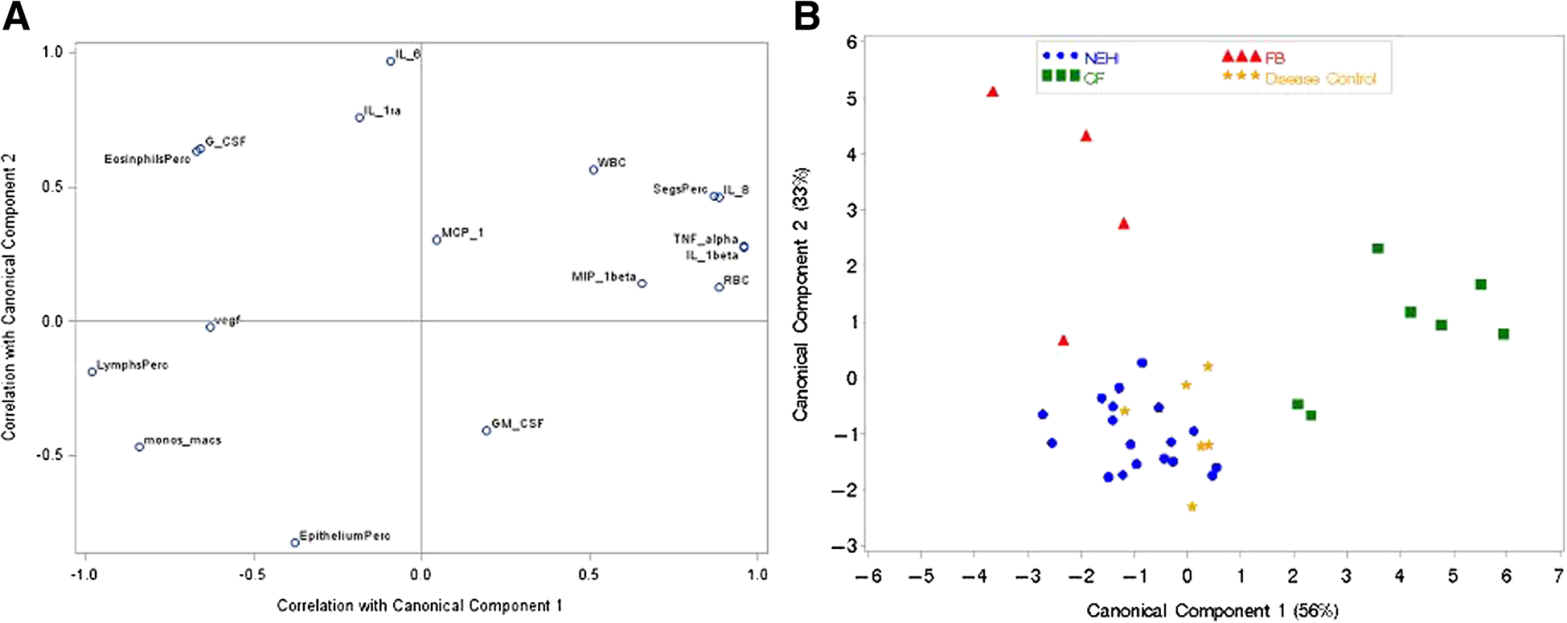
**Results from canonical discriminant analysis.** Canonical Discriminant Analysis is a multivariate method similar to Principal Component Analysis (PCA) yet differs in that we have prior knowledge of the groups. **A)** displays the correlation between each BALF measurement and the first two components which cumulatively explain 89% of the group differences. This displays the weights attributed to each BALF variable which are used to calculate the scores for each sample. **B)** A plot of the first two component score values for each individual identified by disease group. The first two components result in good separation between disease groups, the CF and FB groups are distinguished from the NEHI and disease controls which look similar.

CDA was used to estimate linear combinations of all CBC and cytokine measurements which best discriminated between the four disease groups. The first two components cumulatively explained 89% of the group differences. The first component corresponds to the linear combination which explains most of the variability across groups, the second component explains most of the variability that remains after the first component, and so on. The magnitude of the factor loadings (correlations with components) for each variable indicate how much weight that variable was given in the component. The first component was highly positively correlated with IL-1β, TNF-α, number of red blood cells (RBC), IL-8 and percentage of Neutrophils (Segs %) (Figure 3A). Higher values of these variables correspond to higher component values which are associated with CF (Figure 3B). Percentage of lymphocytes (Lymphs %), percentage of Monocytes and Macrophages (monos_macs), percentage of eosinophils (EOS %), G-CSF and VEGF were highly negatively correlated with the first component and higher values for these variables were associated with FB. The second component had a strong positive correlation with IL-6, and a strong negative correlation with percentage of epithelial cells (epithelium %). This second component separated the FB and CF groups from the NEHI and DC groups, where FB and CF had higher IL-6 and NEHI and DC had a higher percentage of epithelium cells (Figure 3A, 3B). All patient samples were correctly classified into their corresponding disease groups by cross-validation.

## Discussion

ChILD diseases are rare, frequently difficult to diagnose and create hardships for children and their families. A high index of suspicion and a complete evaluation are needed to correctly identify these diseases, as they often have similar signs and symptoms at presentation like NEHI and FB. Although no deaths have been reported from NEHI, NEHI patients may require oxygen use for years and aggressive nutritional supplementation. Glucocorticoid therapy has not been found to be effective and currently is not routinely recommended [4,5]. At our center, NEHI is one of the most common chILD diseases treated. Other chILD disorders can result in serious morbidity, including recurrent pulmonary exacerbations, respiratory failure with prolonged mechanical ventilation, lung transplantation, pulmonary hypertension, and/or prolonged oxygen requirements. Patients in this more severe group are routinely treated aggressively with glucocorticoids and other immunosuppressive agents in addition to supportive care [5,18]. Thus, a correct diagnosis is critical to optimize and direct the treatment plan and provide guidance for the family. New approaches to improve diagnostic strategies are needed. Though preliminary in nature, our pilot data suggests biomarkers may be identified in BALF and helpful in distinguishing NEHI and FB from other airway diseases.

A specific chILD diagnosis commonly requires lung biopsy in patients with chronic or severe symptoms, although the invasiveness of the procedure, physical discomfort, surgical risks and need for hospitalization after the procedure has increased interest in less invasive diagnostic modalities. Recently, patients with NEHI syndrome have been diagnosed using clinical findings, CT scan, infant pulmonary functions and negative findings on bronchoscopy in some centers [19]. However, despite gains in recognizing the classic clinical phenotype of NEHI, some children present with atypical clinical phenotypes that may require lung biopsy. There is also little understanding of the underlying disease mechanism and limited therapeutic options to address years of morbidity for children and their families with NEHI. Our data suggest that the BALF in NEHI is significantly less inflammatory and distinctly different than other types of chILD diseases that predominately involve the airways. These results would be consistent with NEHI lung biopsy findings that show a paucity of inflammation, in contrast to FB and other airways diseases, and could reinforce the clinical practice of limiting daily glucocorticoid and other immunomodulatory treatments in NEHI.

BALF samples from disease groups all contained potential bacterial pathogens with the CF group containing the highest percent. Though NEHI had positive bacterial cultures, the cell counts in the BALF did not appear inflammatory in nature. Furthermore one NEHI patient without evidence of BALF inflammation had *Pseudomonas aeruginosa* (non mucoid) and *Serratia marcescens* cultured from the BALF. It may be that unlike CF patients who had evidence of significant neutrophilia on BALF, NEHI patients did not activate a response in the lower airways with these bacteria or the majority of these bacteria were from the upper airways.

Pulmonary neuroendocrine cells (PNECs) are innervated cells located in the airways that are postulated to have roles in lung development, oxygen sensation, dyspnea, inflammation, bronchoconstriction and vasodilatation [20]. They may increase in in other conditions besides NEHI, including Bronchopulmonary dysplasia, Cystic Fibrosis, Sudden Infant Death and Asthma [2,9]. They are also known to produce chemically active amines and peptides such as serotonin (5-HT), Bombesin/Gastrin releasing peptide (GRP), calcitonin gene-related peptide (CGRP), Substance P (SP) and neuronal markers including neuron specific enolase (NSE) [21]. Thus, we strongly hypothesize that biomarker signals other than pro-inflammatory cytokines and chemokines may be distinguishing and provide more insight into the pathogenesis of NEHI. These studies are clearly warranted using more advanced discovery genomic and proteomic approaches with expanded panels of proteins to investigate BALF and serum. Though our current study lacked the ability to measure these bioactive PNEC substances, our data suggest that these substances are not activating a pro-inflammatory cytokine response in NEHI. Elucidating disease mechanisms involved in NEHI may also be important to further our understanding in other diseases associated with PNEC abnormalities.

Within the adult interstitial lung disease (ILD) literature, several groups have detailed different BALF cellular profiles showing increased lymphocytosis in Non- specific Interstitial Pneumonitis (NSIP) and Cryptogenic Organizing Pneumonia (COP) compared to Usual Interstitial Pneumonia (UIP); in contrast, neutrophils were elevated in UIP in comparison to NSIP and COP [14,22]. Cytokine and chemokine profiles have also been reported in BALF of adult ILD patients to include elevations of IL-ra, VEGF, IL-8, ENA-78 levels compared to controls. A group of eight proteins has recently been propose as disease progression serum markers in Idiopathic Pulmonary Fibrosis (IPF): KL-6, surfactant protein A, and MMP-7, CCL-18, S100A12, IL-8, ICAM-1 and VCAM-1 [14,18,22-25]. Unfortunately, studies of BALF and serum biomarkers in children with chILD lag behind. No BALF cytokine data exists in the chILD literature, although Fan and colleagues recently published their use of serum KL-6 in differentiating NEHI from patients with surfactant dysfunction mutations [26]. Unfortunately, the KL-6 assay is not commercially available in the United States. Our results add to the literature as the first to examine BALF cytokines and chemokines in chILD.

The finding of increased inflammatory cytokines in FB, specifically IL-6, MCP-1, and IL-1ra may highlight the role of these cytokines in the formation of the germinal centers near the airway that characterize FB. Lymphocytic disorders of the lung, such as FB, have been proposed to have an immune or autoimmune etiology, though cases in children are frequently idiopathic at the time of presentation [13]. The association of IL-6 with autoimmune disease like rheumatoid arthritis and transplantation rejection may lend credence to this as a potential disease pathway in FB [27]. Furthermore, distinguishing FB from NEHI is also clinically relevant as some in the chILD community speculate that NEHI and FB may be from the same spectrum of disease and clinically patients can look very similar. Finally, IL-6 may be a potential therapeutic target as humanized anti–IL-6 receptor antibody, tocilizumab, has been used to target the IL-6 pathway in rheumatoid arthritis, Castleman disease, and systemic lupus erythematosus [27]. Further studies are indicated of the IL-6 pathway and chILD, especially those with a potential autoimmune etiology.

The BALF results in our CF group were consistent with previous publications showing elevations in WBCs, predominately neutrophils, and increased levels of tumor necrosis factor (TNF)- α, interleukin (IL)-1β, IL-6, IL-8, granulocyte macrophage colony–stimulating factor (GM-CSF), and granulocyte colony– stimulating factor (GCSF) in airway secretions compared to controls [28]. Recently elevated levels of CC chemokines MCP-1(CCL2), MIP-1α,(CCL3), MIP-1β and (CCL4) and MIP-3α (CCL20) were reported in BALF of young children with CF with little apparent lung disease or infection [29]. Our data also shows elevations in MIP-1β and MCP-1 in our small sample of CF subjects with limited BALF infections.

This study has inherent limitations related to small sample size, retrospective study design, and biased patient selection from a single center where not all patients with these diagnoses underwent BAL due to the clinical situation for each subject. However, in rare disease, small sample sizes to evaluate pilot data are common before large sample collection from multiple institutions can be obtained to further validate preliminary findings. Despite these limitations, the NEHI samples set of 22 BALF is one of the largest samples of patients with NEHI published and this data represents the first set of distinguishing cytokine measured in chILD.

## Conclusions

This study demonstrates the potential value of BALF biomarker analysis for diagnosis and insight into disease pathogenesis for chILD, especially NEHI and FB. Further study is needed to validate these findings and extend our BALF studies using more advanced genomic and proteomic molecular technology approaches.

## Abbreviations

ABCA: ATP- binding cassette transporter; BAL: Bronchoalveolar lavage; BALF: Bronchoalveolar lavage fluid; CDA: Canonical discriminant analysis; CF: Cystic fibrosis; chILD: Children’s interstitial lung disease; DC: Disease control; EDTA: Ethylenediaminetetra acetic acid; FB: Follicular bronchiolitis; FTT: Failure to thrive; GGO: Ground-glass opacity; G-CSF: Granulocyte colony - stimulating factor; GM-CSF: Granulocyte macrophage colony - stimulating factor; GGO: Ground-glass opacity; HRCT: High-resolution computed tomography; IL: Interleukin; ILD: Interstitial lung disease; IQR: Interquartile range; MIP-1: Macrophage inflammatory protein 1; NEB: Neuroepithelial body; NEHI: Neuroendocrine cell hyperplasia of infancy; NICU: Neonatal intensive care unit; PIG: Pulmonary interstitial glycogenosis; PMSF: Phenylmethylsulfonyl fluoride; PNEC: Pulmonary neuroendocrine cell; RBC: Red blood cells; SIDS: Sudden infant death syndrome; SP: Surfactant protein; TNF-α: Tumor necrosis factor alpha; TTF: Thyroid transcription factor; VEGF: Vascular endothelial growth factor; WBC: White blood cell.

## Competing interests

The following authors disclose that they have no financial interests in the subject of this manuscript: J Popler, BD Wagner, H Luckey Tarro, FJ Accurso, RR Deterding.

## Authors’ contributions

JP, FA, RD carried cytokine analysis and were responsible for manuscript preparation. BW completed statistical analysis and assisted with manuscript preparation. HT assisted with patient and data collection. All authors read and approved the final manuscript.
